# Shiga toxin-converting phages and the emergence of new pathogenic *Escherichia coli*: a world in motion

**DOI:** 10.3389/fcimb.2014.00080

**Published:** 2014-06-20

**Authors:** Rosangela Tozzoli, Laura Grande, Valeria Michelacci, Paola Ranieri, Antonella Maugliani, Alfredo Caprioli, Stefano Morabito

**Affiliations:** European Reference Laboratory for Escherichia coli, Dipartimento di Sanità Pubblica Veterinaria e Sicurezza Alimentare, Istituto Superiore di SanitàRome, Italy

**Keywords:** *Escherichia coli*, Shigatoxin, *stx*-phages, STEC, pathogroups

## Abstract

Shiga toxin (Stx)-producing *Escherichia coli* (STEC) are pathogenic *E. coli* causing diarrhea, hemorrhagic colitis (HC) and hemolytic uremic syndrome (HUS). STEC are characterized by a constellation of virulence factors additional to Stx and have long been regarded as capable to cause HC and HUS when possessing the ability of inducing the attaching and effacing (A/E) lesion to the enterocyte, although strains isolated from such severe infections sometimes lack this virulence feature. Interestingly, the capability to cause the A/E lesion is shared with another *E. coli* pathogroup, the Enteropathogenic *E. coli* (EPEC). In the very recent times, a different type of STEC broke the scene causing a shift in the paradigm for HUS-associated STEC. In 2011, a STEC O104:H4 caused a large outbreak with more than 800 HUS and 50 deaths. Such a strain presented the adhesion determinants of Enteroaggregative *E. coli* (EAggEC). We investigated the possibility that, besides STEC and EAggEC, other pathogenic *E. coli* could be susceptible to infection with s*tx*-phages. A panel of *stx2*-phages obtained from STEC isolated from human disease was used to infect experimentally *E. coli* strains representing all the known pathogenic types, including both diarrheagenic *E. coli* (DEC) and extra-intestinal pathogenic *E. coli* (ExPEC). We observed that all the *E. coli* pathogroups used in the infection experiments were susceptible to the infection. Our results suggest that the *stx2*-phages used may not have specificity for *E. coli* adapted to the intestinal environment, at least in the conditions used. Additionally, we could only observe transient lysogens suggesting that the event of stable *stx2*-phage acquisition occurs rarely.

## Introduction

The ability to produce Shiga toxins (Stx) is the major virulence feature of Shiga toxin-producing *Escherichia coli* (STEC). These potent cytotoxins block the protein synthesis by inactivating ribosomes (Okuda et al., 2006) and their action on the target cells is responsible for the most severe forms of STEC-induced disease, such as hemorrhagic colitis and the life-threatening hemolytic uremic syndrome (HUS) (Karmali, 2009). The Stx-coding genes, *stx*, are conveyed by lambdoid bacteriophages (O'brien et al., 1984), which, following infection of a susceptible *E. coli* strain, are maintained into a lysogenic state in the host chromosome, becoming a virulence marker for STEC. In spite of the striking effect exerted by Stx on susceptible cells, their sole production seems to be not sufficient for STEC to induce severe disease in humans. In fact, STEC associated with HUS usually colonize the gastrointestinal tract of the host by inducing a typical lesion to the enterocyte known as attaching and effacing (A/E) (Mcdaniel and Kaper, 1997). Noteworthy, the ability to induce the A/E lesion is shared with Enteropathogenic *E. coli* (EPEC), another *E. coli* pathogroup historically causing outbreaks of infection with high mortality rates in Europe and US up to the end of the second World War, and nowadays representing a leading cause of diarrhea and infant mortality in the developing countries (Tozzoli and Scheutz, 2014). Until recently this combination of virulence traits, the ability to produce Stx and to cause the A/E lesion, represented the paradigm for HUS-associated STEC, altogether defined as Enterohaemorrhagic *E. coli* (EHEC) (Levine, 1987).

In 2011, in Germany, an outbreak of STEC infections occurred that caused more than 4000 infections, including 900 HUS cases and 50 deaths (Bielaszewska et al., 2011). The outbreak strain, beside the production of Stx, possessed the intestinal colonization apparatus typical of another *E. coli* pathogroup, known as Enteroaggregative *E. coli* (EAggEC). As a matter of fact, the outbreak strain possessed the adhesion-associated genes typical of EAggEC such as *aggR*, *aaiC*, *sepA*, *aatA* (Schmidt et al., 1995; Boisen et al., 2008, 2012) and, at the same time, it carried a bacteriophage conveying the genes encoding the Shiga toxin type 2 subtype a (*stx2a*) (Bielaszewska et al., 2011; Scheutz et al., 2011).

The impact of the German outbreak was so deep that the causative STEC strain became iconic of a new *E. coli* pathotype: the Enteroaggregative Hemorrhagic *E. coli* (EAHEC) (Brzuszkiewicz et al., 2011). It has to be noted that *E. coli* strains matching the virulence genes profile of the EAHEC O104:H4 had been previously observed in a few occasions. At the beginning of the 90s, a small HUS outbreak occurred in France (Morabito et al., 1998). The episode was associated to infection with an *E. coli* O111:H2 strain possessing the ability to colonize the host gut by the staked-brick adhesion mechanism typical of EAggEC (Nataro and Kaper, 1998) but which was also able to elaborate Stx2 (Morabito et al., 1998). Additionally, after the German outbreak caused by EAHEC O104:H4, a few sporadic cases of infection with similar EAHEC strains were retrospectively described in the time period 2000–2010 (Iyoda et al., 2000; Scavia et al., 2011). Finally, an HUS case, associated with EAHEC O111:H21 and an outbreak of EAHEC O127:H4 infections occurred, in Northern Ireland in 2012 (Dallman et al., 2012) and in Italy in 2013 (unpublished), respectively.

Up to the present day, four different EAHEC serotypes have been therefore identified including the O111:H2 (Morabito et al., 1998), O104:H4 (Bielaszewska et al., 2011), O111:H21 (Dallman et al., 2012), and O127:H4. This observation, together with the reported isolation of *Enterobacteriaceae* other than *E. coli* producing Stx from cases of human disease (Tschape et al., 1995; Paton and Paton, 1996) suggests that *stx*-phages can infect a range of bacterial hosts wider than expected. Further evidences supporting this hypothesis had been provided by Schmidt and co-workers, who were able to infect and lysogenize different *E. coli* pathogroups including EPEC, STEC, EAggEC, and EIEC using a chloramphenicol-resistant derivative of an Stx2-encoding Bacteriophage (Schmidt et al., 1999).

Recently the susceptibility of EAggEC strains of different serotypes to infection with the stx2-phage P13374 obtained from the German EAHEC O104:H4 strain has been investigated (Beutin et al., 2012). In that study, however, all the strains tested were found to be resistant to a high infective dose of P13374 and the authors concluded that the phage used had a restricted host range among EAggEC.

In order to contribute additional evidences to the subject matter, we investigated the ability of a panel of six *stx2*-phages to infect and lysogenize *E. coli* strains belonging to all the know pathogroups, including both diarrheagenic (DEC) and extraintestinal pathogenic *E. coli* (ExPEC). We describe that, at least in the conditions used in the laboratory, all the phages used were able to infect some of the strains belonging to all the *E. coli* pathogroups. Additionally, one of the phages used was able to produce lysogens visible after one cultural passage, which were no longer observable at the following passage.

Our observations suggest that the *stx*-phages used have a broad host specificity toward different *E. coli* pathogroups but that their stable acquisition might be a rare event.

## Materials and methods

### Bacterial strains and *stx2*-phages

All the strains used in the present study are part of the collections held at Istituto Superiore di Sanità. A total of 33 *E. coli* strains were used as recipients for infection experiments. In detail, five typical Enteropathogenic *E. coli* (tEPEC), five atypical EPEC strains (aEPEC), five Enteroaggregative *E. coli* (EAggEC), five Enterotoxigenic *E. coli* (ETEC), three Enteroinvasive *E. coli* (EIEC), five Extraintestinal Pathogenic *E. coli* (ExPEC) strains isolated from urinary tract infections and five non-pathogenic *E. coli* strains from the ECOR collection (Ochman and Selander, 1984) were infected with *stx2*-phages. Typical and atypical EPEC were positive to PCR specific for the intimin-coding *eae* gene (Oswald et al., 2000). All typical EPEC also possessed the EAF plasmid as assessed by PCR of the BfP-coding gene (Franke et al., 1994).

All the EAggEC possessed the *aat* (Schmidt et al., 1995), *aggR* and *aaiC* (Boisen et al., 2012) genes as assessed by PCR in the conditions described in the respective papers.

PCR amplification was also used to identify the presence of the heat-stable (ST) and heat-labile (LT) enterotoxins coding-genes (Liu et al., 2013), *ipaH* (Liu et al., 2013) and the genes encoding the cytotoxic-necrotizing factor (CNF) (Kadhum et al., 2006) to verify the pathogroups ETEC, EIEC and ExPEC, respectively, in the conditions indicated in the respective papers.

*E. coli* K12 strains LE392 and DH5α have been used as control strains in infection experiments and for the *stx2*-phages amplification.

The *E. coli* strains CB553/5 and C125-06 (courtesy of Dr. Flemming Scheutz) and ED 191, ED 924, ED 703 and ED 508, were used to obtain the *stx2*-phages used for the infections by the spot agar assays. The CB553/5 strain is an ETEC O166:H15 possessing both *stx2* and LT genes and isolated from a human case of non-complicated diarrhea in Denmark. Strain C125-06 is a STEC O103:H25 isolated during a large HUS outbreak occurred in Norway in 2006 (Schimmer et al., 2008), possessing an *stx2*-phage with the gene encoding the Stx2 B subunit interrupted by an IS*1203* (this study, data not shown), which doesn't produce an active Shiga toxin. Strains ED 191 (O111:H2) and ED 924 (O127:H4) are Enteroaggregative-Hemorrhagic *E. coli* (EAHEC) strains isolated from small HUS outbreaks occurred in France in 1992 (Morabito et al., 1998) and in Northern Italy in 2013 (unpublished), respectively. Strain ED 703 is an O104:H4 EAHEC isolated from a HUS case occurred in Italy in 2009 (Scavia et al., 2011). Strain ED 508 is a STEC O157 producing Stx2 and isolated in Italy from a human case of disease. The *stx2*-phages derived from these *E. coli* strains have been termed with the strain's name preceded by the prefix Phi (e.g., Phi-508 from strain ED 508).

The panel of six Stx2-converting phages used to set up the infection experiments was selected using as a criterion their association with STEC of public health relevance. As a matter of fact, all the *stx*-phages conveyed the Stx2-coding genes, the toxin type associated with the most severe forms of STEC infections. Additionally, phages conveying such Stx type are the only ones so far associated with *E. coli* pathogroups other than the classical STEC.

### Phages induction and amplification

The *E. coli* strains carrying the *stx2*-phages were exposed to UV light in order to induce the excision of the phage genome from the bacterial chromosome (Sambrook and Russell, 2001). In detail, each bacterial strain was grown in Luria-Bertrani (LB) broth (Oxoid Limited, Basingstoke Hampshire, UK) overnight at 37°C with vigorous shaking. The culture was diluted 1:100 in LB modified broth (LB with 0.001% thiamine V/V) grown to 0.5 OD 600 and centrifuged. The bacterial pellets were re-suspended in a sterile solution of CaCl_2_ 10 mM. The culture was exposed to UV light (130 μ Joule × 100) in a “Stratalinker^®^ UV crosslinker” (Stratagene Cloning Systems, La Jolla, CA, USA). After induction, the culture was diluted in LB modified broth and incubated at 37°C for 5 h with vigorous shaking. The culture was centrifuged and the supernatant containing phages particles filtered with 0.45 μm pore-filters. 100 μl of phage particles suspension were added to 100 μl of a culture of the propagator strain *E. coli* LE392 grown in LB modified broth at 0.5 OD 600 and the resulting mixture was maintained at 37°C for 20 min with static incubation. Each tube was added with 3.5 ml of LB modified soft agar (LB modified broth with agar 7 g/L) kept at 42°C and immediately poured on LB modified agar plates (LB modified broth with agar 15 g/L). Plates were incubated overnight at 37°C.

Four ml of SM buffer (100 mM NaCl, 8 mM MgSO_4_·7H_2_O, 50 mM Tris-HCl 1 M pH 7.5, Gelatin 0.002%) were dispensed to each plate in order to recover phages particles from the lytic plaques and kept overnight at 4°C. The phage suspension in SM was recovered and added with chloroform at 5% final concentration. The phage suspension has been centrifuged at 500× g 10 min twice for removing agar debris and used to re-infect the propagator *E. coli* strain LE392 in the conditions described above in order to increase the phage titre. Finally, the phage suspension was concentrated by using Amicon Ultra-15 Centrifugal Filter Unit with Ultracel-30 tubes (Merck Millipore, Billerica, MA, USA) with a cut-off of 30 KDa. Final phage titres ranged from 1 × 10^10^ to 4.9 × 10^11^ PFU/ml.

### Determination of the *stx2*-phages integration sites in the *E. coli* genome

The *stx2*-phages integration sites in the *E. coli* strains were determined. The occupancy of loci *sbcB*, *wrbA*, *yehV*, and Z2577 was assessed as previously described (Serra-Moreno et al., 2007). An additional primer pair (YecE_fwd GCTAGCGCCGAGCAGCACAA/YecE_rev ATGGCCGATGGCACCTGTCT) was deployed for specifically investigating the integrity of the locus *yecE* (this study).

### Shigatoxin-genes subtyping

The determination of *stx*-genes subtypes was performed by PCR as previously described (Scheutz et al., 2012).

### Plaque lift and hybridization

Plaque blot experiments were performed to assay the homogeneity of the phages suspensions (Sambrook and Russell, 2001). In detail, phage induction experiments were performed as described above. A nylon membrane (Hybond N^+^, GE Healthcare Life Sciences, UK) was placed onto the surface of each agar plate containing the phages plaques originated by lysis of the propagator strain and kept in place for 5 min. The filters were lifted from the plates and incubated with denaturation buffer (1.5 M NaCl, 0.5 M NaOH) at room temperature for 5 min and dried on a Whatman^®^ paper 3 mm (GE Healthcare Life Sciences, UK). Filters were transferred to a new Whatman^®^ paper 3 mm sheet impregnated with neutralization buffer (0.5 M Tris-Cl pH 7.2, 1 M NaCl) for 5 min. The membranes were dried and incubated with SSC 2× (6 M NaCl and 0.6 M sodium citrate) for 5 min. Once dried, 1 μl of a positive control, made up by unlabeled DNA corresponding to the probe used in the following hybridizations, was spotted on each filter. DNA was fixed by UV light (1200μ Joule × 100) in a crosslinker “Stratalinker^®^ UV crosslinker” (Stratagene Cloning Systems, La Jolla, CA, USA).

A DNA fragment obtained by PCR amplification of the s*tx2*-gene using the primers Stx2F/Stx2R (Paton and Paton, 2002) was labeled by incorporating the digoxygenin-11-deoxy-uridine-triphosphate using the PCR DIG probe synthesis Kit (Roche Diagnostics, Switzerland) and used as a probe. A pre-hybridization step was performed incubating the membranes in a buffer containing SSC 5×, 0.01% N-Lauryl Sarcosine, 1% blocking reagent (Roche Diagnostics, Switzerland) at 68°C for 1 h. The hybridization was carried out by incubating the filters overnight with 500 ng of the labeled probe at 68°C. The membranes were washed in SSC 2× with 0.1% SDS two times with agitation for 5 min followed by two washes in SSC 0.04× with 0.1% SDS at 64°C 15 min. The detection of the hybridized Digoxygenin-labeled probe was carried out with Anti- Digoxigenin AP Fab fragments and the detection reagent NBT/BCIP solution following the manufacturer's instructions (Roche Diagnostics, Switzerland).

### Infection experiments by spot agar assay

The susceptibility of *E. coli* strains to the *stx2*-phages was assessed by spot agar assay as described elsewhere (Muniesa et al., 2004). In detail, each host strain was grown in LB medium overnight with vigorous shaking, diluted 100 times in LB modified broth (LB with 0.001% thiamine V/V) and grown at 0.5 OD 600. One hundred μl of the culture were added with 4 ml of LB modified soft agar (LB modified broth with agar 7 g/L) at 42°C and immediately poured on LB modified agar plates (LB modified broth with 15 g/L agar). Ten μl of each *stx2*-phage was spotted on each recipient strain immediately after the solidification of the soft agar layer. Phage titres used were: 1 × 10^10^ PFU/ml for the *stx2*-phages Phi- C125-06, Phi-703 and Phi-191, 1.5 × 10^10^ PFU/ml for Phi-CB553/5, 1.9 × 10^10^ PFU/ml for Phi-508 and 4.9 × 10^11^ for the *stx2*-phage from the EAHEC ED 924, Phi-924. Plates were incubated overnight at 37°C.

### Assessment of toxin production and colony blot

In all the cases where lytic areas were not visible, the infection of the recipient strains was verified by carrying out Vero cell assays for the identification of the Stx production (Caprioli et al., 1994).

In detail, part of the top agar taken from the plates in correspondence with the area where the s*tx*2-phage suspension was applied during the spot agar assay, was incubated in 2 ml of Tripticase Soya Broth (TSB, Oxoid Limited, Basingstoke Hampshire, UK) overnight at 37°C. One ml of the culture was centrifuged at 13000×g 10 min and the supernatant was filtered with 0.45 μm pore-filters. Twenty μl of each supernatant were inoculated on a semi-confluent monolayer of Vero cells. The presence of cythopathic effect was assessed at 24 h and confirmed at 48 and 72 h.

The *E. coli* recipients infected with the *stx*2-phage Phi-C125-06:IS*1203*, which does not induce the production of an active Stx, were verified by colony blot. Scalar dilutions of the overnight cultures obtained as described above were diluted and plated on LB agar plates in way of having about 50 colonies on each of the plates.

Bacterial colonies were transferred to a nylon membrane with high affinity for nucleic acids (Hybond N^+^, GE Healthcare Life Sciences, UK) by keeping the filter in contact with the surface of the agar for 5 min. The filters were incubated with denaturation buffer (1.5 M NaCl, 0.5 M NaOH) for 20 min and dried on a Whatman^®^ paper 3 mm (GE Healthcare Life Sciences, UK). A further incubation on a Whatman^®^ paper 3 mm impregnated with a buffer containing Triton X-100 0.2 % and NaOH 0.05 N was carried out for 10 min. Once dried, each filter was moved to a new sheet of Whatman^®^ paper 3 mm impregnated with neutralization buffer (0.5 M Tris-Cl pH 7.2, 1 M NaCl) and dried again. The hybridization and detection steps were carried out as described above in the section “Plaque Lift and Hybridization.”

## Results

### Characterization of the *stx2*-phages used in the infection experiments

Although all the phages used in the infection experiments were obtained from epidemiologically unrelated *E. coli* strains with different genomic background, including STEC, EAggEC and ETEC, they have been partially characterized to ascertain that we were not dealing with a homogeneous phage population made up by identical phage types.

Recently a peculiar type of *stx2*-phage has been described as being associated with STEC O157 isolated from human infections (Tozzoli et al., 2014). Such a phage, termed Φ8, presented large genomic differences when compared to the sequence of the reference *stx2*-phage BP933W, present in the reference STEC O157 strain EDL933, in the regions where the Stx-coding genes are located and containing the genes regulating the switch between lysogeny and lytic cycle, respectively (Tozzoli et al., 2014). All the phages in the panel used in the infection experiments were negative to a Φ8-specific PCR (Tozzoli et al., 2014) but the Phi-508 phage, indicating that only the latter belonged to this particular phage type.

The whole genome sequences (WGS) of the strains CB553/5, ED 508, ED 703 and ED 924 and of the phage Phi-191 from the EAHEC O111:H2 that caused the outbreak in France in the 90s have been determined (data not shown). The contigs composing the WGS of the bacterial strains were mapped against the genomic sequence of the phage Phi-191 (data not shown).

The comparison showed that in the genomes of the three EAHEC strains were present stx2-phages showing an average similarity with the Phi-191 of 90% and higher, indicating that the three phages belonged to a homogeneous population. Conversely, the contigs of the ETEC strain CB553/5 only mapped in a small region of 7750 bp containing the genes encoding the Stx2, indicating that, in this strain the *stx2*-phage must be extensively different from that present in the EAHEC strains.

The sequence of the phage the phage Phi-C125-06 corresponds to the published sequence of phage TL-2011c, isolated from a STEC O103:H25 during an outbreak of HUS cases occurred in Norway in 2006 (Schimmer et al., 2008). We found that the sequence of phage Phi-191 was about 90% homologous to that of TL-2011c (data not shown), similarly to how reported for the phage P13374, present in the EAHEC O104:H4 that caused the German outbreak in 2011 (Beutin et al., 2012). Additionally, in the Phi-C125-06 an IS 1203 was detected in the gene encoding the B subunit of the Stx.

Finally, we observed that the contigs composing the WGS of the strain ED 508 that mapped on the sequence of the phage Phi-191 shared an average 65% of homology at nucleotide level.

Taken together these findings indicate that the stx2-phages used in the infection experiments belonged to at least three different types.

The PCR sub-typing scheme deployed by Scheutz and coworkers was used to identify the subtypes of the Shiga toxin-coding genes harbored by the *stx2*-phages used in this study (Scheutz et al., 2012). All the phages harbored *stx2*_*a*_ subtype, with the exception of Phi-CB553/5, which possessed *stx2*_*d*_.

The insertion sites in the parental bacterial strains were determined for all the *stx2*-phages but the Phi-C125-06, which was already described to be present in the *wrbA* locus (Sekse et al., 2008a). With the exception of Phi-508, which is localized in *yehV* locus, all the other phages composing the set used in this study seemed to be inserted in *wrbA*.

The homogeneity of the phage suspensions used in the experimental infections was determined by infecting the propagator K 12 strain LE 392 and by performing plaque lift and hybridization with a probe corresponding to the *stx2* gene encoding the Stx2 A subunit. All the plaques present on the plates were positive to hybridization with the *stx2* probe, indicating that the all the phage suspensions used in the following infection experiments contained a unique bacteriophage population.

### Susceptibility of *E. coli* strains to the infection with the *stx2*-phages

*E. coli* strains belonging to all the known pathotypes were infected with the set of six *stx2*-phages. The results of the spot agar tests are reported in Table 1. In total, we assayed the susceptibility of a panel of 35 *E. coli* strains, including five typical EPEC (tEPEC, BfP-positive), five atypical EPEC (aEPEC, BfP-negative), five ETEC, five EAggEC strains, three EIEC, five ExPEC, five *E. coli* non-pathogenic strains belonging to the ECOR collection (Ochman and Selander, 1984) and two *E. coli* K 12 laboratory strains.

**Table 1:** **Results of the spot agar assays showing the susceptibility of the *E. coli* strains to infection with the different *stx2*-phages used**.

**Pathogroup (No. of strains)**	**No. of strains showing lysis upon infection with Stx2-Phages**					
	**Phi-C125-06**	**Phi-703**	**Phi-191**	**Phi-508**	**Phi-924**	**Phi-CB553/5**
EIEC (3)	1	3	3	3	2	3
ExPEC (5)	0	0	0	0	0	5
tEPEC (5)	0	1	1	0	1	5
aEPEC (5)	2	0	3	1	1	5
ETEC (5)	0	1	1	5	0	5
EAggEC (5)	1	0	2	1	1	5
ECOR collection (5)	1	3	4	2	2	5
*E. coli* K 12 (2)	2	2	2	2	2	2
Tot. (35)	7	10	16	14	9	35

With the exception of the bacteriophage obtained from the STEC/ETEC strain (CB553/5), which induced visible lysis in all the strains infected, all the other phages were able to induce clear lysis halos only in *E. coli* strains belonging to the Diarrheagenic *E. coli* (DEC) pathogroups, although with different efficiency (Table 1), but not in ExPEC cultures. Finally, the two *E. coli* K 12 strains used as controls demonstrated to be susceptible to infection with all the phages tested, showing marked lytic areas.

These results suggested that the *stx2*-phages used in this study, with the exception of the Phi-CB553/5, might have a specificity for DEC.

To ascertain if the lack of lytic areas in the negative *E. coli* cultures was due to mechanisms of resistance to infection, further analyses were carried out aiming at determining the production of Stx. Such an approach was based on the assumption that, for the toxin to be produced, the phage DNA must be inserted into the chromosome of the bacterial host and the host itself must therefore be susceptible to infection, also in the absence of visible lysis. The occurrence of infection in the absence of lytic areas has been previously reported during experimental infection of a panel of diarrheagenic *E. coli* with a derivative chloramphenicol-resistant *stx2*-phage (Schmidt et al., 1999).

All the bacterial strains used in the experimental infections have been assayed for the capability to induce a cythopathic effect (CPE) onto Vero cells monolayers before infection with the *stx2*-phages and most of them proved negative by microscopic observation of the cell up to 72 h post inoculum. Of the 35 *E. coli* strains assayed two EXPEC, one tEPEC, two aEPEC, one EAggEC, and four ETEC strains induced the death of Vero cells and have therefore been excluded from the following screening by Vero cells assay (VCA).

The bacterial strains that gave negative results in the VCA and that, at the same time, did not show lysis in the spot agar assay, were tested again after the experimental infection with the phages by inoculating the supernatant of bacterial cultures obtained by incubating in a liquid medium the area of the plates where the phage suspension was applied on the test *E. coli* strains, onto Vero cell monolayers.

By using this approach all the cultures showed a cytopathic effect (CPE). The CPE observed appeared after 24 h incubation and progressed toward the complete death of the monolayer in 48–72 h.

An alternative strategy was adopted to verify if the lack of lytic areas in the test strains infected with phage Phi-C125-06 was due to mechanisms of resistance. As a matter of fact, this phage doesn't possess an intact Stx2 B subunit-coding gene and therefore would not induce any CPE on Vero cells monolayers. The bacterial cultures derived by inoculating the area of the spot agar test where the phage was applied were plated onto solid media and subjected to colony blot using a probe corresponding to a region of the gene encoding the Stx2 A subunit. None of the colonies were positive in the hybridization experiments, suggesting that the strains had not been infected.

### Verification of the stable acquisition of *stx2*-phages by the *E. coli* strains

Following the observation that *E. coli* strains belonging to all the pathogroups used were susceptible to infection with the *stx2*-phages used, we investigated on the possibility that the *E. coli* strains were able to maintain the phage DNA stably integrated in their chromosome. Sub-cultures of the infected strains that produced Stx were diluted and analyzed by colony blot to verify the presence of the *stx2* gene in their genome. Lysogens were only observed in the cultures of one ETEC and one EAggEC strains as well as in the *E. coli* K 12 strain LE 392 used as control, all infected with the Phi 191 phage. The latter lysogen was stable and could be cultured several times, while the cultures from the pathogenic *E. coli* became negative already at the second cultural passages after the phage infections.

## Discussion

STEC are human pathogens whose complex nature and pathogenicity mechanisms are not completely understood yet. As a matter of fact, a number of different geno-phenotypes have been described so far in STEC strains isolated from human infections displaying a wide and diverse range of symptoms including mild diarrheal disease as well as life threatening forms such as the HUS (Tozzoli and Scheutz, 2014). The association between the production of Stx and the ability to cause the A/E lesion, has been long regarded as the virulence features asset characterizing the STEC causing HUS (Levine, 1987), with the understood feeling that the virulence gene repertoires described in other STEC had to be associated with the less severe forms of the infection. This approach led to the definition of schemes attempting at categorizing STEC for the purpose of laying the ground for a proper epidemiological approach and management of infections. The most comprehensive among those schemes was developed by Karmali and co-workers a decade ago (Karmali et al., 2003) and considered either the clinical aspects of the infection or the virulence features of the STEC strains. Such a scheme used the term seropathotype (SPT) to define the different groups of STEC, with the SPTs A and B including those causing the most severe forms of infection or associated with outbreaks (Karmali et al., 2003). Notably, the STEC included in the SPTs A and B are all capable to cause the A/E lesion.

This reference scheme has been efficaciously used to frame the STEC isolated from human disease for many years. Nevertheless, in 2011, one of the largest and most severe outbreaks of STEC infection occurred in Germany and France (Frank et al., 2011; Mariani-Kurkdjian et al., 2011) and caused the paradigm to vault in a new direction. The infecting strain, a STEC O104:H4 was undoubtedly associated with HUS, with an impressive proportion of cases of infection progressing toward this severe syndrome (Frank et al., 2011) and, at the same time, it was not able to induce the A/E lesion (Bielaszewska et al., 2011).

The investigation on the genetic asset of the outbreak strain revealed that beside the ability to produce Stx, it possessed the virulence genes encoding the adhesion machinery of Enteroaggregative *E. coli* (EAggEC) (Bielaszewska et al., 2011) and it has been proposed that this strain belongs to a new pathogroup of *E. coli* termed Enteroaggeregative Haemorrhagic *E. coli* (EAHEC) (Brzuszkiewicz et al., 2011). Several hypothesis have been made on the evolution of the EAHEC O104:H4 including the opposite views of the derivation of this strain from an EAggEC that acquired an *stx2*-phage by horizontal gene transfer (Brzuszkiewicz et al., 2011; Rasko et al., 2011) or proposing its evolution from an ancestor STEC O104:H4 by stepwise gain and loss of chromosomal and plasmid-encoded virulence factors (Mellmann et al., 2011). More recently evidences have been provided showing that the EAHEC O104:H4 could have evolved by the uptake of a *stx2*-phage originated from the bovine reservoir by an EAggEC O104:H4 (Beutin et al., 2013). This scenario parallels what it can be inferred for the STEC strains causing the attaching and effacing lesion, such as those belonging to SPTs A and B (Karmali et al., 2003) which are also termed Enterohemorrhagic *E. coli* (EHEC) (Levine, 1987). As a matter of fact, the latter can be considered as atypical EPEC (aEPEC) that developed the capability to produce Stx (Trabulsi et al., 2002). As in the case of EAggEC and EAHEC, aEPEC and EHEC share the same mechanism of colonization and are mainly distinguished by the capability to produce Stx, which increase their pathogenicity.

The above considerations bring into question if STEC are indeed an *E. coli* pathogroup in its own right, or rather if this pathogen represents multiple pathogroups whose virulence has been increased by the event of an *stx*-phage acquisition. Or even if the phage itself might be considered as being the pathogen, using the *E. coli* colonization machinery to establish a successful infection in the final host. Whatever scenario is chosen, it is undeniable that for any intestinal infection to be successful, a pathogen must efficiently colonize the mucosa, overcoming the competition with the resident microflora. In this respect, both the SPTs A and B STEC and EAHEC can rely on efficient colonization machineries, with the one present in the latter being recognized as causing the most diffuse and long-lasting intestinal colonization (Nataro and Kaper, 1998). Accordingly, while both the groups have been associated with HUS, the EAHEC O104:H4 infections during the German outbreak of 2011 progressed toward this severe form in about 30% of cases (Frank et al., 2011), against the typical 5%–10% of cases of infection with SPT A and B STEC strains developing HUS (Tarr et al., 2005).

How stated above admits the possibility that any *E. coli* strain could acquire the ability to produce Stx and that, virtually any pathogenic *E. coli* might stem a stable augmented-pathogenicity variant threatening the public health systems. This is hold true for EAggEC, which beside the German outbreak have been described as being associated with HUS at least in other two outbreaks occurred in the 90s in France (Morabito et al., 1998) and in 2013 in Italy (unpublished) as well as in some unrelated cases recorded in Europe and Asia in the period 2000–2012 (Iyoda et al., 2000; Scavia et al., 2011; Beutin et al., 2012; Dallman et al., 2012).

In the conditions used in our laboratory we observed that, beside the EAggEC, other *E. coli* pathogroups were susceptible to infection with different *stx2*-phages obtained from a number of different STEC types including EAHEC, typical STEC O157 and non-O157 as well as from an hybrid ETEC/STEC strain isolated from a patient with diarrhea in Denmark (Dr. F. Scheutz, personal communication). All the phages assayed conveyed the genes encoding the Stx2 type (predominantly Stx2a subtype), the one associated with STEC causing HUS and the only one found in Stx-producing EAggEC so far.

We ascertained that in the conditions used in the laboratory, all the DEC types assayed, including tEPEC, aEPEC, EAggEC, ETEC, EIEC, were susceptible to infection with the *stx2*-phages. Additionally, the *stx2*-phage from the hybrid ETEC/STEC strain CB553/5 infected all the *E. coli* strains tested, including ExPEC, and the field isolates of non-pathogenic *E. coli* of the ECOR collection.

We observed that the *stx2*-phages used in this study seem not to have specificity for particular *E. coli* groups although the efficacy of the infections varied with the phage used (Table 1).

Our results are in line with the identification of the Stx2-producing ETEC described above, and with the recent report of a case of septicemia in a human patient with evidence of infection with an *E. coli* strain possessing the genes encoding the Stx2 and matching the genetic background of an ExPEC (Wester et al., 2013). Our results are also in agreement with how reported in a previous study on the transduction of a chloramphenicol-resistant *stx2*-phage where *E. coli* strains belonging to all the diarrheagenic *E. coli* groups were successfully infected and produced stable lysogens (Schmidt et al., 1999). These findings provide evidences that the *stx2*-phage acquisition can involve a spectrum of *E. coli* hosts wider than expected and that no pathogroup-specific barriers seem to exist to the acquisition of *stx2*-phages belonging to the types used in this work.

However, it cannot be excluded that *stx*-phages different from those used in this study might have a restricted host range and that in the natural environment the spreading of the *stx*-phages to *E. coli* hosts might be hindered by factors related with the bacterial hosts. The existence of such restrictions could explain how reported in a recent study where the susceptibility of a panel of 31 EAggEC to infection with the *stx2*-phage derived from the EAHEC O104:H4 that caused the German outbreak in 2011 was assessed (Beutin et al., 2012). The authors observed that none of the bacterial strains tested showed evidences of infection with the *stx2*-phage in spite of the high titer used (Beutin et al., 2012).

Differences in the host range associated with the phage type could be also hypothesized by considering the results of the spot agar tests performed in this study. As a matter of fact, we observed that while the phage Phi-CB553/5 induced clear lysis in all the strains analyzed, all the other phages infected a variable number of strains in each of the pathogroups but never induced lysis in the ExPEC strains analyzed. Nevertheless we could observe that such strains induced CPE onto Vero cells monolayers after the infection with the *stx2*-phages. This observation, although not confirmed by sero-neutralization, suggests a wider host range of the phages used in this study. Further work is needed to clarify the mechanisms leading to the selection of the *stx*-phages able to infect the different *E. coli* pathogroups.

Interestingly, all the *E. coli* pathotypes, with the exception of typical STEC, have a human reservoir and an inter-human transmission cycle (Nataro and Kaper, 1998), while the typical STEC and the related *stx*-phages seem to have their reservoir in the intestinal tract of ruminants (Caprioli et al., 2005; Beutin et al., 2013). This observation implies that for the event of an *stx*-phage acquisition by a human *E. coli* to occur a common ecosystem must exist where the two organisms may encounter and interact. Such an ecosystem could be represented by the intestine of a mammalian host or the environment (Schmidt et al., 1999; Sekse et al., 2008b; Imamovic et al., 2009; Dopfer et al., 2010). Given the inter-human circulation of the EAggEC, the possibility that EAHEC may have arisen following an environmentally mediated event of an *stx2*-phage acquisition is interesting. As a matter of fact in low-income countries, where infections with DEC are endemic the ineffective treatments of human sewages may account for a wide dispersion of these pathogens in the environment, with the consequent possibility for them to come into contact with *stx*-phages originating from ruminant's excreta. Accordingly, an origin in these countries has been traced or postulated for some of the Stx2-producing EAggEC (Scavia et al., 2011; Beutin et al., 2012; Weiser et al., 2013), strengthening such a hypothesis for their derivation.

## Author contributions

Rosangela Tozzoli conceived the experimental design and drafted the manuscript, Laura Grande carried out the experimental work and analysis, and revised critically the manuscript, Paola Ranieri carried out part of experiments (stx subtyping and insertion sites identification), Valeria Michelacci and Antonella Maugliani participated in the revision of the manuscript and supported Laura Grande in the experimental work, Alfredo Caprioli contributed to critical the revision of the draft manuscript for important intellectual content, Stefano Morabito conceived the study and strongly contributed to revise the manuscript. Finally, all the authors approved the manuscript to be published.

### Conflict of interest statement

The authors declare that the research was conducted in the absence of any commercial or financial relationships that could be construed as a potential conflict of interest.

## References

[B1] Beutin L. Hammerl J. A. Reetz J. Strauch E. (2013). Shiga toxin-producing *Escherichia coli* strains from cattle as a source of the Stx2a bacteriophages present in enteroaggregative *Escherichia coli* O104:H4 strains. Int. J. Med. Microbiol. 303, 595–602 10.1016/j.ijmm.2013.08.001 24012149

[B2] Beutin L. Hammerl J. A. Strauch E. Reetz J. Dieckmann R. Kelner-Burgos Y. (2012). Spread of a distinct Stx2-encoding phage prototype among *Escherichia coli* O104:H4 strains from outbreaks in Germany, Norway, and Georgia. J. Virol. 86, 10444–10455 10.1128/JVI.00986-12 22811533 PMC3457275

[B3] Bielaszewska M. Mellmann A. Zhang W. Kock R. Fruth A. Bauwens A. (2011). Characterisation of the *Escherichia coli* strain associated with an outbreak of haemolytic uraemic syndrome in Germany, 2011: a microbiological study. Lancet Infect. Dis. 11, 671–676 10.1016/S1473-3099(11)70165-7 21703928

[B4] Boisen N. Scheutz F. Rasko D. A. Redman J. C. Persson S. Simon J. (2012). Genomic characterization of enteroaggregative *Escherichia coli* from children in Mali. J. Infect. Dis. 205, 431–444 10.1093/infdis/jir757 22184729 PMC3256949

[B5] Boisen N. Struve C. Scheutz F. Krogfelt K. A. Nataro J. P. (2008). New adhesin of enteroaggregative *Escherichia coli* related to the Afa/Dr/AAF family. Infect. Immun. 76, 3281–3292 10.1128/IAI.01646-07 18443096 PMC2446688

[B6] Brzuszkiewicz E. Thurmer A. Schuldes J. Leimbach A. Liesegang H. Meyer F. D. (2011). Genome sequence analyses of two isolates from the recent *Escherichia coli* outbreak in Germany reveal the emergence of a new pathotype: entero-aggregative-haemorrhagic *Escherichia coli* (EAHEC). Arch. Microbiol. 193, 883–891 10.1007/s00203-011-0725-6 21713444 PMC3219860

[B7] Caprioli A. Luzzi I. Rosmini F. Resti C. Edefonti A. Perfumo F. (1994). Community-wide outbreak of hemolytic-uremic syndrome associated with non-O157 verocytotoxin-producing *Escherichia coli*. J. Infect. Dis. 169, 208–211 10.1093/infdis/169.1.208 8277184

[B8] Caprioli A. Morabito S. Brugere H. Oswald E. (2005). Enterohaemorrhagic *Escherichia coli*: emerging issues on virulence and modes of transmission. Vet. Res. 36, 289–311 10.1051/vetres:2005002 15845227

[B9] Dallman T. Smith G. P. O'brien B. Chattaway M. A. Finlay D. Grant K. A. (2012). Characterization of a verocytotoxin-producing enteroaggregative *Escherichia coli* serogroup O111:H21 strain associated with a household outbreak in Northern Ireland. J. Clin. Microbiol. 50, 4116–4119 10.1128/JCM.02047-12 23035193 PMC3502983

[B10] Dopfer D. Sekse C. Beutin L. Solheim H. Van Der Wal F. J. De Boer A. (2010). Pathogenic potential and horizontal gene transfer in ovine gastrointestinal *Escherichia coli*. J. Appl. Microbiol. 108, 1552–1562 10.1111/j.1365-2672.2009.04575.x 19863689

[B11] Frank C. Werber D. Cramer J. P. Askar M. Faber M. An Der Heiden M. (2011). Epidemic profile of Shiga-toxin-producing *Escherichia coli* O104:H4 outbreak in Germany. N. Engl. J. Med. 365, 1771–1780 10.1056/NEJMoa1106483 21696328

[B12] Franke J. Franke S. Schmidt H. Schwarzkopf A. Wieler L. H. Baljer G. (1994). Nucleotide sequence analysis of enteropathogenic *Escherichia coli* (EPEC) adherence factor probe and development of PCR for rapid detection of EPEC harboring virulence plasmids. J. Clin. Microbiol. 32, 2460–2463 7814482 10.1128/jcm.32.10.2460-2463.1994 PMC264083

[B13] Imamovic L. Jofre J. Schmidt H. Serra-Moreno R. Muniesa M. (2009). Phage-mediated Shiga toxin 2 gene transfer in food and water. Appl. Environ. Microbiol. 75, 1764–1768 10.1128/AEM.02273-08 19168651 PMC2655461

[B14] Iyoda S. Tamura K. Itoh K. Izumiya H. Ueno N. Nagata K. (2000). Inducible stx2 phages are lysogenized in the enteroaggregative and other phenotypic *Escherichia coli* O86:HNM isolated from patients. FEMS Microbiol. Lett. 191, 7–10 10.1111/j.1574-6968.2000.tb09311.x 11004392

[B15] Kadhum H. J. Ball H. J. Oswald E. Rowe M. T. (2006). Characteristics of cytotoxic necrotizing factor and cytolethal distending toxin producing *Escherichia coli* strains isolated from meat samples in Northern Ireland. Food Microbiol. 23, 491–497 10.1016/j.fm.2005.07.003 16943042

[B16] Karmali M. A. (2009). Host and pathogen determinants of verocytotoxin-producing *Escherichia coli*-associated hemolytic uremic syndrome. Kidney Int. Suppl. S4–S7 10.1038/ki.2008.608 19180132

[B17] Karmali M. A. Mascarenhas M. Shen S. Ziebell K. Johnson S. Reid-Smith R. (2003). Association of genomic O island 122 of *Escherichia coli* EDL 933 with verocytotoxin-producing *Escherichia coli* seropathotypes that are linked to epidemic and/or serious disease. J. Clin. Microbiol. 41, 4930–4940 10.1128/JCM.41.11.4930-4940.2003 14605120 PMC262514

[B18] Levine M. M. (1987). *Escherichia coli* that cause diarrhea: enterotoxigenic, enteropathogenic, enteroinvasive, enterohemorrhagic, and enteroadherent. J. Infect. Dis. 155, 377–389 10.1093/infdis/155.3.377 3543152

[B19] Liu J. Gratz J. Amour C. Kibiki G. Becker S. Janaki L. (2013). A laboratory-developed TaqMan Array Card for simultaneous detection of 19 enteropathogens. J. Clin. Microbiol. 51, 472–480 10.1128/JCM.02658-12 23175269 PMC3553916

[B20] Mariani-Kurkdjian P. Bingen E. Gault G. Jourdan-Da Silva N. Weill F. X. (2011). *Escherichia coli* O104:H4 south-west France, June 2011. Lancet Infect. Dis. 11, 732–733 10.1016/S1473-3099(11)70266-3 21958580

[B21] Mcdaniel T. K. Kaper J. B. (1997). A cloned pathogenicity island from enteropathogenic *Escherichia coli* confers the attaching and effacing phenotype on E. coli K-12. Mol. Microbiol. 23, 399–407 10.1046/j.1365-2958.1997.2311591.x 9044273

[B22] Mellmann A. Harmsen D. Cummings C. A. Zentz E. B. Leopold S. R. Rico A. (2011). Prospective genomic characterization of the German enterohemorrhagic *Escherichia coli* O104:H4 outbreak by rapid next generation sequencing technology. PLoS One 6:e22751 10.1371/journal.pone.0022751 21799941 PMC3140518

[B23] Morabito S. Karch H. Mariani-Kurkdjian P. Schmidt H. Minelli F. Bingen E. (1998). Enteroaggregative, Shiga toxin-producing *Escherichia coli* O111:H2 associated with an outbreak of hemolytic-uremic syndrome. J. Clin. Microbiol. 36, 840–842 9508328 10.1128/jcm.36.3.840-842.1998 PMC104641

[B24] Muniesa M. Serra-Moreno R. Jofre J. (2004). Free Shiga toxin bacteriophages isolated from sewage showed diversity although the stx genes appeared conserved. Environ. Microbiol. 6, 716–725 10.1111/j.1462-2920.2004.00604.x 15186350

[B25] Nataro J. P. Kaper J. B. (1998). Diarrheagenic *Escherichia coli*. Clin. Microbiol. Rev. 11, 142–201 9457432 10.1128/cmr.11.1.142 PMC121379

[B26] O'brien A. D. Newland J. W. Miller S. F. Holmes R. K. Smith H. W. Formal S. B. (1984). Shiga-like toxin-converting phages from *Escherichia coli* strains that cause hemorrhagic colitis or infantile diarrhea. Science 226, 694–696 10.1126/science.6387911 6387911

[B27] Ochman H. Selander R. K. (1984). Standard reference strains of *Escherichia coli* from natural populations. J. Bacteriol. 157, 690–693 6363394 10.1128/jb.157.2.690-693.1984 PMC215307

[B28] Okuda T. Tokuda N. Numata S. Ito M. Ohta M. Kawamura K. (2006). Targeted disruption of Gb3/CD77 synthase gene resulted in the complete deletion of globo-series glycosphingolipids and loss of sensitivity to verotoxins. J. Biol. Chem. 281, 10230–10235 10.1074/jbc.M600057200 16476743

[B29] Oswald E. Schmidt H. Morabito S. Karch H. Marches O. Caprioli A. (2000). Typing of intimin genes in human and animal enterohemorrhagic and enteropathogenic *Escherichia coli*: characterization of a new intimin variant. Infect. Immun. 68, 64–71 10.1128/IAI.68.1.64-71.2000 10603369 PMC97102

[B30] Paton A. W. Paton J. C. (1996). Enterobacter cloacae producing a Shiga-like toxin II-related cytotoxin associated with a case of hemolytic-uremic syndrome. J. Clin. Microbiol. 34, 463–465 8789041 10.1128/jcm.34.2.463-465.1996 PMC228823

[B31] Paton A. W. Paton J. C. (2002). Direct detection and characterization of Shiga toxigenic *Escherichia coli* by multiplex PCR for stx1, stx2, eae, ehxA, and saa. J. Clin. Microbiol. 40, 271–274 10.1128/JCM.40.1.271-274.2002 11773130 PMC120136

[B32] Rasko D. A. Webster D. R. Sahl J. W. Bashir A. Boisen N. Scheutz F. (2011). Origins of the E. coli strain causing an outbreak of hemolytic-uremic syndrome in Germany. N. Engl. J. Med. 365, 709–717 10.1056/NEJMoa1106920 21793740 PMC3168948

[B33] Sambrook J. Russell D. (ed.). (2001). Molecular Cloning: a Laboratory Manual. New York, NY: Cold Spring Harbor Laboratory Press

[B34] Scavia G. Morabito S. Tozzoli R. Michelacci V. Marziano M. L. Minelli F. (2011). Similarity of Shiga toxin-producing *Escherichia coli* O104:H4 strains from Italy and Germany. Emerg. Infect. Dis. 17, 1957–1958 10.3201/eid1710.111072 22000382 PMC3310690

[B35] Scheutz F. Nielsen E. M. Frimodt-Moller J. Boisen N. Morabito S. Tozzoli R. (2011). Characteristics of the enteroaggregative Shiga toxin/verotoxin-producing *Escherichia coli* O104:H4 strain causing the outbreak of haemolytic uraemic syndrome in Germany, May to June 2011. Euro Surveill. 16 Available online at: http://www.eurosurveillance.org/viewarticle.aspx?articleid=19889 10.2807/ese.16.24.19889-en 21699770

[B36] Scheutz F. Teel L. D. Beutin L. Pierard D. Buvens G. Karch H. (2012). Multicenter evaluation of a sequence-based protocol for subtyping Shiga toxins and standardizing Stx nomenclature. J. Clin. Microbiol. 50, 2951–2963 10.1128/JCM.00860-12 22760050 PMC3421821

[B37] Schimmer B. Nygard K. Eriksen H. M. Lassen J. Lindstedt B. A. Brandal L. T. (2008). Outbreak of haemolytic uraemic syndrome in Norway caused by stx2-positive *Escherichia coli* O103:H25 traced to cured mutton sausages. BMC Infect. Dis. 8:41 10.1186/1471-2334-8-41 18387178 PMC2335110

[B38] Schmidt H. Bielaszewska M. Karch H. (1999). Transduction of enteric *Escherichia coli* isolates with a derivative of Shiga toxin 2-encoding bacteriophage phi3538 isolated from *Escherichia coli* O157:H7. Appl. Environ. Microbiol. 65, 3855–3861 10473386 10.1128/aem.65.9.3855-3861.1999 PMC99711

[B39] Schmidt H. Knop C. Franke S. Aleksic S. Heesemann J. Karch H. (1995). Development of PCR for screening of enteroaggregative *Escherichia coli*. J. Clin. Microbiol. 33, 701–705 7751380 10.1128/jcm.33.3.701-705.1995 PMC228017

[B40] Sekse C. Muniesa M. Wasteson Y. (2008a). Conserved Stx2 phages from *Escherichia coli* O103:H25 isolated from patients suffering from hemolytic uremic syndrome. Foodborne Pathog. Dis. 5, 801–810 10.1089/fpd.2008.0130 19014273

[B41] Sekse C. Solheim H. Urdahl A. M. Wasteson Y. (2008b). Is lack of susceptible recipients in the intestinal environment the limiting factor for transduction of Shiga toxin-encoding phages? J. Appl. Microbiol. 105, 1114–1120 10.1111/j.1365-2672.2008.03845.x 18492045

[B42] Serra-Moreno R. Jofre J. Muniesa M. (2007). Insertion site occupancy by stx2 bacteriophages depends on the locus availability of the host strain chromosome. J. Bacteriol. 189, 6645–6654 10.1128/JB.00466-07 17644594 PMC2045183

[B43] Tarr P. I. Gordon C. A. Chandler W. L. (2005). Shiga-toxin-producing *Escherichia coli* and haemolytic uraemic syndrome. Lancet 365, 1073–1086 10.1016/S0140-6736(05)71144-2 15781103

[B44] Tozzoli R. Grande L. Michelacci V. Fioravanti R. Gally D. Xu X. (2014). Identification and characterization of a peculiar vtx2-converting phage frequently present in Verocytotoxin-producing Escherichia coli (VTEC) O157 isolated from human infections. Infect. Immun. [Epub ahead of print]. 10.1128/IAI.01836-14 24799627 PMC4097640

[B45] Tozzoli R. Scheutz F. (2014). Diarrhoeagenic *Escherichia coli* infections in humans, in Pathogenic Escherichia Coli, Molecular and Cellular Microbiology, 1st Edn., ed. Stefano M. (Norfolk: Caister Academic Press), 1–18

[B46] Trabulsi L. R. Keller R. Tardelli Gomes T. A. (2002). Typical and atypical enteropathogenic *Escherichia coli*. Emerg. Infect. Dis. 8, 508–513 10.3201/eid0805.010385 11996687 PMC2732489

[B47] Tschape H. Prager R. Streckel W. Fruth A. Tietze E. Bohme G. (1995). Verotoxinogenic Citrobacter freundii associated with severe gastroenteritis and cases of haemolytic uraemic syndrome in a nursery school: green butter as the infection source. Epidemiol. Infect. 114, 441–450 10.1017/S0950268800052158 7781732 PMC2271295

[B48] Weiser A. A. Gross S. Schielke A. Wigger J. F. Ernert A. Adolphs J. (2013). Trace-back and trace-forward tools developed ad hoc and used during the STEC O104:H4 outbreak 2011 in Germany and generic concepts for future outbreak situations. Foodborne Pathog. Dis. 10, 263–269 10.1089/fpd.2012.1296 23268760 PMC3698685

[B49] Wester A. L. Brandal L. T. Dahle U. R. (2013). Extraintestinal pathogenic *Escherichia coli* carrying the Shiga Toxin gene stx2. J. Clin. Microbiol. 51, 4279–4280 10.1128/JCM.01349-13 24108605 PMC3838058

